# Biased Signaling of CCL21 and CCL19 Does Not Rely on N-Terminal Differences, but Markedly on the Chemokine Core Domains and Extracellular Loop 2 of CCR7

**DOI:** 10.3389/fimmu.2019.02156

**Published:** 2019-09-13

**Authors:** Astrid S. Jørgensen, Olav Larsen, Edith Uetz-von Allmen, Michael Lückmann, Daniel F. Legler, Thomas M. Frimurer, Christopher T. Veldkamp, Gertrud M. Hjortø, Mette M. Rosenkilde

**Affiliations:** ^1^Department of Biomedical Sciences, Faculty of Health and Medical Sciences, University of Copenhagen, Copenhagen, Denmark; ^2^Biotechnology Institute Thurgau (BITg), University of Konstanz, Kreuzlingen, Switzerland; ^3^The Novo Nordisk Foundation Center for Basic Metabolic Research, University of Copenhagen, Copenhagen, Denmark; ^4^Department of Chemistry, University of Wisconsin-Whitewater, Whitewater, WI, United States

**Keywords:** ligand biased signaling, CCR7, CCL21, CCL19, chemokine core domain, ECL2

## Abstract

Chemokine receptors play important roles in the immune system and are linked to several human diseases. Targeting chemokine receptors have so far shown very little success owing to, to some extent, the promiscuity of the immune system and the high degree of biased signaling within it. CCR7 and its two endogenous ligands display biased signaling and here we investigate the differences between the two ligands, CCL21 and CCL19, with respect to their biased activation of CCR7. We use *bystander bioluminescence resonance energy transfer* (BRET) based signaling assays and Transwell migration assays to determine *(A)* how swapping of domains between the two ligands affect their signaling patterns and *(B)* how receptor mutagenesis impacts signaling. Using chimeric ligands we find that the chemokine core domains are central for determining signaling outcome as the lack of β-arrestin-2 recruitment displayed by CCL21 is linked to its core domain and not N-terminus. Through a mutagenesis screen, we identify the extracellular domains of CCR7 to be important for both ligands and show that the two chemokines interact differentially with extracellular loop 2 (ECL-2). By using *in silico* modeling, we propose a link between ECL-2 interaction and CCR7 signal transduction. Our mutagenesis study also suggests a lysine in the top of TM3, K130^3.26^, to be important for G protein signaling, but not β-arrestin-2 recruitment. Taken together, the bias in CCR7 between CCL19 and CCL21 relies on the chemokine core domains, where interactions with ECL-2 seem particularly important. Moreover, TM3 selectively regulates G protein signaling as found for other chemokine receptors.

## Introduction

The chemokine receptor CCR7 and its two endogenous ligands, CCL21 and CCL19, are involved in the lymph node homing of several T cell subpopulations and antigen-presenting dendritic cells (DC), which is central for the DC priming of T cells for antigen-specific activation (1). The system is an example of ligand bias (2, 3), where the two agonists elicit differential signaling patterns through the same receptor. Two other forms of bias exist, namely receptor bias and tissue bias (3, 4). At CCR7, CCL19 and CCL21 both induce G protein signaling, whereas only CCL19 induces β-arrestin-2 recruitment leading to receptor internalization and recycling (5–7). CCL21, on the other hand, has been found to induce much stronger ERK phosphorylation than CCL19 (7).

Chemokine receptors belong to the large G protein-coupled receptor (GPCR) superfamily, a diverse group in which the receptors respond to a variety of ligand types and are involved in a wide range of physiological processes. Chemokines are 8–12 kD peptides and larger than the ligands of many other GPCRs (8). In contrast to small molecules, which can bind very deep in the binding pocket (9), chemokines mainly interact with the extracellular domains of the receptor (8). Since GPCRs play a central role in so many different physiological systems they constitute a highly interesting group of therapeutic targets, this is why a lot of research focuses on the understanding of their activity. Thus, the receptor family makes up a large part of the drug targets for approved drugs and compounds currently undergoing clinical investigation (10, 11). Where GPCRs at first were perceived to either adopt an active or inactive conformation, it is now widely accepted that the receptors are much more dynamic, existing in several intermediate states and that several distinct active conformations exist each leading to distinct functional outcomes depending on the ligand it interacts with (12–16). Studies have identified distinct areas selectively important for G protein signaling (17–19) or β-arrestin-2 recruitment (20, 21). These studies indicate that individual receptor domains might couple to different signaling pathways independently (4). Thus, targeting of GPCRs can potentially become more effective by targeting the signaling events underlying a specific physiological effect while limiting unwanted side effects. Agonist interactions leading to receptor activation has also been studied in great detail (22) and information of the molecular interactions between one of several agonists with a given receptor could provide a foundation for the manipulation of receptor signaling in a therapeutic setting.

From crystal structures and mutagenesis studies it seems that the chemokine N-terminus enters the receptor by interactions with TM2 in the minor binding pocket [delimited by TM1-3 and−7 (23)], where several central activation initiation interaction residues have been identified (23–26). However, although potentially entering the receptor by a common entry site, different chemokines show diverse utilization of the binding pocket, with some filling the pocket completely (26) and others showing a confinement to the minor binding pocket only (27, 28). Such differential utilization of the binding pocket may also apply for the two ligands interacting with CCR7, for which we have previously identified mutations in the major binding pocket affecting only CCL21-induced, but not CCL19-induced G protein signaling (7).

Studies from the 1990's, covering chemokine—receptor interactions, led to the proposition of a so-called two-step activation model describing how a chemokine interacts with its receptor in both a temporal and functional (two-step) and a spatial (two-site) manner (8, 24, 29–31). The first step consist of interactions between the chemokine core domain and the extracellular domains of the receptor, referred to as chemokine recognition site 1 (CRS1), in a process which is thought to confer high affinity without inducing receptor activation. Instead, the receptor activation takes place during the second step; this often includes interactions of the flexible chemokine N-terminus with the transmembrane domains of the receptor, a site referred to as second chemokine recognition site (CRS2). More recent functional studies and novel crystal structures show that this two-step model is too simple to explain the complex chemokine receptor activation mechanism and several additional interaction sites have been presented and found relevant for chemokine receptor activation (24, 32). However, both the original two-step activation model and its newer updated versions along with studies of chemokine N-termini (33) all suggest that the chemokine N-terminus is central for receptor activation.

In the current study, we investigate the role of the N-terminus and the core domains of CCL21 and CCL19 with regard to the differential signaling pattern of the two chemokines in CCR7 activation. Moreover, we describe CCR7 regions of selective importance for both CCL19 and CCL21. Finally, we identify a region in CCR7 of selective importance for G protein signaling, but not β-arrestin-2 recruitment. Altogether these observations pave the way for future selective targeting of not only one of many chemokines interacting with the same receptor, but also for selective drugs targeting one among a subset of signaling pathways initiated upon agonist binding to its receptor.

## Materials and Methods

### Human Chemokines

The human chemokines CCL19 (catalog No. 361-MI) and CCL21 (catalog No. 366-6C) were purchased from R&D Systems (Minneapolis, MN, USA) or PeproTech (LuBioScience, Luzern, Switzerland).

### Expression and Purification of the Human Chimeric CCL19^CCL21N-term^ and CCL21^CCL19N-term^ Chemokines

The CCL21^CCL19N−term^ chimera was expressed and purified as described in detail in Jørgensen et al. (34), while the CCL19^CCL21N−term^ chimera was expressed and purified as described in detail for the chemokine CCL19 in Veldkamp et al. (35). The CCL21^CCL19N−term^ chimera contains residues 1–16 of human CCL19 and residues 17–111 of human CCL21 resulting in the protein sequence below:

GTNDAEDCCLSVTQKPIPAKVVRSYRKQEPSLGCSIPAILFLPRKRSQAELCADPKELWVQQLMQHLDKTPSPQKPAQGCRKDRGASKTGKKGKGSKGCKRTERSQTPKGP.

The CCL19^CCL21N−term^ chimera consists of residues 1–16 of human CCL21 in place of the same numbered residues in human CCL19 resulting in the protein sequence below.

SDGGAQDCCLKYSQRKIPGYIVRNFHYLLIKDGCRVPAVVFTTLRGRQLCAPPDQPWVERIIQRLQRTSAKMKRRSS.

As described in Veldkamp et al. (36) protein identity was verified by mass spectrometry and one-dimensional NMR spectra of the chimeras were consistent with that of a folded chemokine.

### Mutagenesis

The human CCR7 wild-type cDNA was cloned from a spleen-derived cDNA library. Mutations were introduced into the CCR7 WT pcDNA 3.1^+^ expression vector by PCR using the QuickChange™ site-directed mutagenesis kit according to the manufacturer's instructions (Stratagene, La Jolla, CA). All mutations were verified by DNA sequence analysis.

### Cell Culturing and Transfection

CHO-k1 cells were grown at 5% CO_2_ at 37°C in RPMI supplemented with 10% FBS, 180 U/ml penicillin, and 45 μg/ml streptomycin. Transient transfection of cells used in the BRET assays was performed using the Lipofectamine® 2000 method (Invitrogen) as described by the manufacturers. Briefly, 500,000 cells were seeded in a 6-well plate 1 day before transfection. Cells were transfected with 1 μg DNA in a ratio of 1:5 receptor:Camyel reporter construct (37), for the cAMP accumulation assay, whereas cells used in the β-arrestin-2 recruitment assay were transfected with 330 ng receptor construct, 42 ng of a rLuc8 Arrestin3 construct (a renilla luciferase variant fused to β-arrestin-2) and 800 ng of Membrane Citrulline-YFP constructs. A total of 6 μl Lipofectamine® 2000 was used per well.

Murine 300–19 pre-B cells were grown in RPMI1640 with Ultra-Glutamine supplemented with 10% FCS, 50 μM β-mercaptoethanol (Gibco; LuBioScience, Luzern, Switzerland), 1% v/v Pen/Strep, and 1% non-essential amino acids (BioWhittaker Lonza; VWR Scientific, Nyon, Switzerland). A total of 3 ×10^6^ 300–19 cells were transfected with 2 μg plasmid DNA using the Amaxa Cell Line Nucleofector Kit V and program M-013 (Lonza) and grown for 2 weeks in the presence of 0.8 mg/ml G418. Bulk cell sorting for high CCR7 surface expression using an APC-conjugated antibody reacting with human CCR7 (R&D Systems; Minneapolis, MN; FAB197A) was performed on a BD FACSAria II cell sorter using the FACSDiva 6 software (BD Biosciences).

### Bioluminescence Resonance Energy Transfer (BRET) Assays for G Protein Signaling and β-Arrestin-2 Recruitment

One day after transfection CHO-k1 cells were washed in PBS before resuspension in 3 mL PBS with glucose (5 mM). Cells were aliquoted [85 μl (without forskolin) or 80 μl (with forskolin for G protein signaling)] in 96-well plates and incubated with coelenterazine (Nanolight technologies), for a final concentration of 5 μM, and kept dark. Ligands were added in various concentrations and incubated for 40 min before BRET signal was detected using an Envision plate reader. When used, forskolin was added 5 min after ligands for a final concentration of 10 μM. The BRET signal was calculated as the ratio between the two detected signals from YFP and Renilla Luciferase:

*BRET ratio* = *YFP (525 nm)/rLuc (480nm)*.

### Surface Expression Analysis

300–19 cells were washed with PBS, incubated for 10 min at RT with monoclonal anti-mouse CD16/CD32 antibody (Fc block) and then stained for 20 min at 4°C with anti-human CCR7-APC in staining buffer (PBS + 0.5% FCS, pH 7.4). Unbound antibody was removed by two washing steps with staining buffer, and SYTOX Blue (Invitrogen; LuBio) was added as a dead cell indicator. All samples were filtered (50 μM Cup Filcons; BD Biosciences), measured with an LSRII flow cytometer using the FACSDiva 6 software, and analyzed with the FlowJo software (BD Biosciences).

### Migration Assay

The capacity of 300–19 cells stably expressing wild-type or mutant CCR7 to migrate in a CCR7-dependent manner was measured by an *in vitro* chemotaxis assay as previously described (38, 39). Briefly, cells (1 ×10^5^, 100 μl) were seeded in the top chambers with 5-μm pore size of the Transwells (Corning Costar; Vitaris). Lower chamber wells contained 600 μl of medium supplied with increasing concentrations of human CCL19 or CCL21 (PeproTech; LuBio) or medium without chemokine (random migration control). The plates were incubated for 3 h at 37°C, 5% CO_2_. Filters were removed and migrated cells in the bottom chamber were collected and acquired for 60 s at high flow rate on an LSRII flow cytometer using the FACSDiva 6 software (BD Biosciences). The percentage of specific migration was calculated by dividing the number of cells migrated to the lower well by the total cell input (100 μl cell suspension directly added to 500 μl medium without chemokine in the lower chamber) multiplied by 100 and subtracting random migration (always <0.4%) to the lower chamber without chemokine present. Non-transfected 300–19 cells were used as a negative control.

### Molecular Modeling

A model of CCR7 was generated using the X-ray crystal structure of CCR5 in complex with CCL5 (PDB 4MBS) (40). The N- and C-termini of CCR7 not covered by the template were not considered during model generation and the structural waters of CCR5 were omitted. The models were built using the Full Model Builder of ICM 3.8-7b (Molsoft L.L.C.) and subsequently refined through 200 steps of all-atom Monte Carlo-minimization.

### Statistical Analysis

LogEC_50_ values were determined by non-linear regression calculated using the GraphPad Prism software, which was also used for all statistical calculations. Statistical significances between dose-response curves were analyzed performing two-way ANOVA followed by a Bonferroni post-test. ^***^p < 0.001, ^**^p < 0.01, and ^*^p < 0.05, ns indicates non-significant differences.

## Results

### Ligand Bias With Distinct Signaling Profiles of CCL21 and CCL19

**Figure 1:**
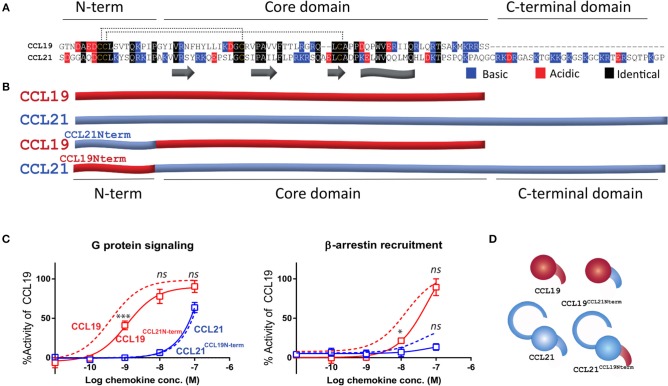
Endogenous CCL21 and CCL19 and chimeric N-terminal swap ligands. **(A)** Sequences of CCL21 and CCL19. The secondary structures are marked below with beta strands identified as arrows and C-terminally alpha helix identified as a sheet. *N-term, core domain*, and *C-terminal domain* refer to domains within the tertiary structure. The 16 N-terminal residues are swapped within **(B)**. Black refers to identical residues in the two sequences, blue refers to positively charged and red refers to negatively charged. **(B)** Schematic overview of CCL21, CCL19, and N-terminally swapped chimeras employed in this study, CCL19^CCL21N−term^ and CCL21^CCL19N−term^. Blue refers to CCL21-derived residues and red to CCL19-derived residues not to be confused with red and blue in **(A)**. **(C)** Dose-response curves of CCL19, CCL21, CCL19^CCL21N−term^, and CCL21^CCL19N−term^ in G protein signaling *(left)* and β-arrestin-2 recruitment *(right)*. The signals are obtained by co-transfecting CHO cells with CCR7 and reporter constructs able to measure intracellular cAMP changes (G protein) or β-arrestin-2 recruitment. Data are measured as the arbitrary unit BRET ratio and normalized to that of CCL19 within each separate experiment to compensate for inter-assay variations. Signaling in response to wild type CCL21 and CCL19 are shown as dotted lines, while the curves of chimeric ligands are shown with solid lines and symbols representing mean values (±SEM). Data are represented as mean values for several independent experiments performed in duplicates (*n* = 7 for G protein signaling and *n* = 6 for β-arrestin-2 recruitment). ANOVA has been used to compare the curves of CCL19 and ^CCL19NCCL21N−term^ or the curves of CCL21 and ^CCL21CCL19N−term^ and asterisks identify significant differences, while *ns* refers to no significant changes. **(D)** Illustrative overview of wild type and chimeric ligand employed in this study with colors as described in **(B)**.

Although selectively acting at the same receptor, the two chemokines CCL21 and CCL19 display a low sequence homology with only 30% sequence identity (Figure 1A). It is therefore interesting to understand how the two chemokines act at the same receptor, but also how they differentiate. Previous studies show that CCL19 is a more potent ligand than CCL21 in both G protein signaling, recruitment of the non-visual arrestins, β-arrestins, as well as in DC migration assays, whereas CCL21 induces a stronger calcium flux and ERK activation (3, 7). Recruitment of β-arrestin-2 toward CCR7 has previously been evaluated using a DiscoverX system (7), where the reporter system relies on fusion proteins consisting of receptor and reporter constructs, but here we reevaluate it using a bystander BRET based assay which relies on the membrane anchoring of YFP and the recruitment of a β-arrestin-2-luciferase fusion protein toward the membrane upon receptor activation. A similar bystander BRET based assay is used to evaluate G protein signaling, namely the cAMP Camyel (37) sensor-based assay which can measure changes of intracellular cAMP as an indicator of e.g., G_α*i*_ activity. By using these two similar assays with the same receptor construct and in the same cell line we are able to avoid any tissue bias that may occur between distinct types of reporter assays tested in different cell types. As expected, CCL21 displays a less potent G protein signal than CCL19, and hardly induces any β-arrestin-2 recruitment (Figure 1C). In contrast, CCL19 stimulates both pathways with higher potencies [logEC_50_ (±SEM) of −9.4 (±0.09) M and −7.9 (±0.10) M], confirming previous studies (7). Based on the suggestions of the N-terminus being central for chemokine signaling we sought to investigate how the two chemokines' N-termini contribute to the differences in signaling profiles.

### G Protein Signaling Is Determined by the Core Domain of the CCR7 Ligands—Not N-Terminus

To evaluate the importance of the chemokine N-terminus of CCL21 and CCL19 with regards to CCR7 signaling we constructed chimeric ligands in which the 16 first N-terminal residues are swapped between the two ligands. The swapped sequences contain both the N-terminus and part of the N-loop, as the N-loop is known to interact with the proximal part of the receptor N-terminus and is important for directing the positioning of the distal chemokine N-terminus. Where CCL21 is more positively charged in both its N-terminus and overall, the N-terminus of CCL19 is more negatively charged (Figure 1A). Swapping of the N-terminal regions gave rise to two chimeric ligands, CCL21^CCL19N−term^ and CCL19^CCL21N−term^ (Figures 1B,D). We first tested the two N-terminal swap chimeras for their ability to activate G protein signaling following CCR7 stimulation through the BRET based cAMP accumulation assay (Figure 1C, left). Where CCL21 induces a weak signal the CCL19 chimera containing the N-terminus of CCL21, CCL19^CCL21N−term^, displayed a fairly strong activation of CCR7, reaching a saturation of the dose-response curve at 100 nM chemokine, which was not significantly different from CCL19, 90% (± 7.6%) compared to 92% (± 4.3%) for CCL19. Performing a *t*-test showed that the chimeric ligand differed slightly from CCL19 with a small but significant decrease of potency with logEC_50_ values (±SEM) of −9.4 (±0.09) M and −8.9 (±0.13) M for CCL19 and CCL19^CCL21N−term^, respectively. In contrast, the CCL21 chimera containing the N-terminus of CCL19, CCL21^CCL19N−term^, induced a weak G protein signal upon CCR7 stimulation, with the dose-response curve actually overlaying that of CCL21, indicating that the N-terminus does not change the signal capacity of CCL21. These data suggest that the G protein signaling is determined by the core domain of the ligands.

### β-Arrestin-2 Signaling Is Similarly Determined by the Core Domain—Not the N-Terminus of the Chemokine

Given that signaling bias at CCR7 is mainly divided into G protein signaling and β-arrestin-2 recruitment, we went on to test the two chimeras' ability to recruit β-arrestin-2. If the signaling bias between the two chemokines resides in the N-terminus, it would be expected that the CCL19^CCL21N−term^ chimera, containing the N-terminus of CCL21 that hardly induces any β-arrestin-2 recruitment, would fail to give a signal. The CCL21^CCL19N−term^ chimera would be expected to stimulate β-arrestin-2 recruitment similar to CCL19. However, this was not the case as the CCL21^CCL19N−term^ chimera failed to induce any β-arrestin-2 recruitment, similar to CCL21, and the CCL19^CCL21N−term^ chimeric ligand induced a strong β-arrestin-2 recruitment resembling the strong signal induced by CCL19 (Figure 1C, right). The chimera reached the same signaling strength at 100 nM chemokine and only seemed to differ from CCL19 with a change of potency—similar to that which we showed for the G protein signal of the chimera. Overall, this suggests that the β-arrestin-2 recruitment is likewise determined by the core domain of the chemokines.

### Differential Docking Modes of CCL21 and CCL19 to CCR7

**Figure 2:**
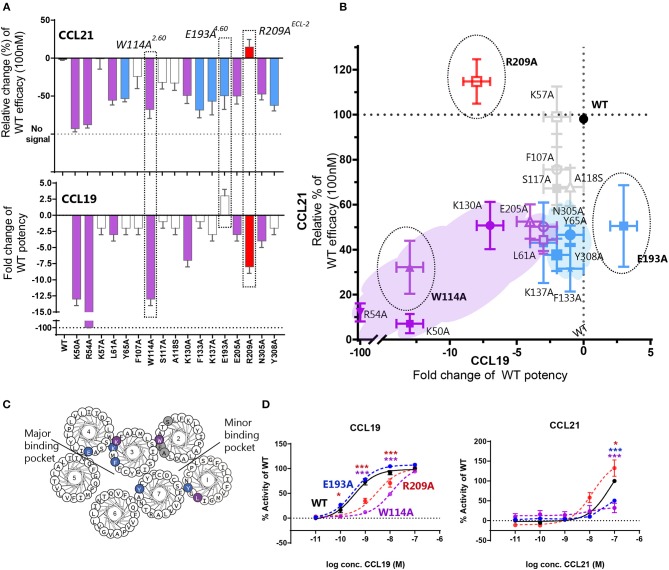
Mutagenesis study of CCR7 showing changes of CCL21 and CCL19 G protein signaling by CCR7 mutations. **(A)** Barplot displaying change of CCL21- or CCL19-signaling by CCR7 mutations evaluated in a cAMP accumulation assay. Changes are displayed as relative change of efficacy at 100 nM CCL21, or fold change of potency for CCL19. Colors correspond to colors in **(B)**, where *purple* identifies mutations impairing both ligands, *blue* refers to mutations only affecting CCL21, *red* refers to mutations only affecting CCL19, and *gray* identifies mutations with no impact. Three mutations are highlighted, which are also highlighted in **(B)** and presented with their dose-response curves in **(D)**. **(B)** Scatterplot comparing the effect on CCL21 signaling to that of CCL19. Mutations are plotted with their values from **(A)** and colored according to the description in **(A)**. **(C)** Helical wheel of CCR7 with mutations identified according to effect on CCR7 signaling in **(A,B)**. **(D)** Dose-response curve of CCR7^W114A^, CCR7 ^E193A^, CCR7 ^R209A^, and CCR7^WT^ stimulated with CCL21 or CCL19 in a cAMP accumulation assay. Significant differences between mutant and WT curve analyzed by two-way ANOVA are identified with colored asterisks corresponding to the color of the signaling curve. Data are represented as mean values (±SEM) of at least three independent experiments performed in duplicates. To compensate for inter-assay variations data have been normalized to wildtype within each separate experiment before the collection of data. The *n* value of independent experiments for each mutation can be found in Table 1.

**Figure 3:**
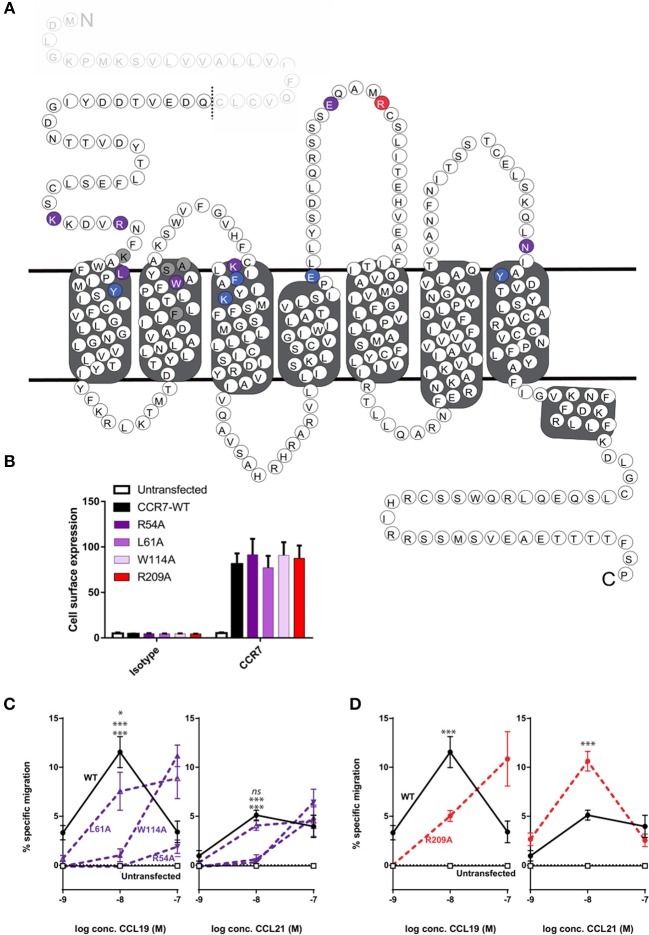
Overview of mutagenesis study, surface expression, and change of CCL21- and CCL19-directed migration by CCR7 mutations. **(A)** Serpentine structure of CCR7 with mutations from Figure 2 identified according to effect on CCR7 signaling, *purple* identifies mutations impairing both ligands, *blue* refers to mutations only affecting CCL21, and *red* refers to mutations only affecting CCL19. Gray shows residue where alanine substitution did not affect signaling. The predicted 24-residues N-terminal signal sequence of CCR7 which is cleaved of from the mature protein (39) is displayed as faded and predicted cleavage site is indicated by a dotted line. **(B)** Surface expression levels of CCR7 were analyzed by flow cytometry in 300–19 pre-B-cells stably transfected with CCR7^WT^, CCR7^R54A^, CCR7^L61A^, CCR7^W114A^, or CCR7^R209A^ constructs using anti-human CCR7-APC (gated on the live cell population). Data show mean APC fluorescence intensity values (±SEM) derived from all migration assays performed in **(C,D)**. **(C,D)** Transwell chemotaxis in response to CCL21 and CCL19 by R54A^N−term^, L61A^1.35^, and W114A^2.60^ **(C)** or R209A^ECL−2^ **(D)**. 300–19 cells were allowed to migrate in response to gradient concentrations of chemokines for 180 min. Migrated cells were counted and percentages of specifically migrated cells relative to the input were calculated. Mean values (±SEM) derived from four independent experiments are shown. Asterisks identify significant differences between WT and the mutant at 10 nM chemokine calculated performing two-way ANOVA. In **(C)** significance levels are positioned with L61A at top, followed by W114A (middle), and R54A (bottom).

To obtain more detailed structural information of the different docking modes of CCL19 and CCL21, we expanded our previously published CCR7 mutation library (7) with 13 mutations more focused on the extracellular domains and the minor binding pocket of CCR7. All mutations were created as alanine substitutions and in parallel with the previously published mutations tested for their impact on CCL21 or CCL19-mediated G protein activation by co-transfecting the mutated CCR7 construct with the cAMP Camyel sensor (Figure 2A and Table 1). Comparing the effect of the mutations on CCL21 and CCL19 signaling (Figures 2B,C) supports the prior findings of a differential docking mode of the two chemokines in CCR7 (7). Accordingly, only CCL21, but not CCL19, seems to depend on interactions within the major binding pocket (F133^3.29^, K137^3.33^, E193^4.60^, and Y308^7.32^). In contrast, both ligands depend on residues found within and around the minor binding pocket, including the proximal part of the N-terminus (K50^N−term^, R54^N−term^, L61^1.35^, and W114^2.60^ important for both ligands and Y65^1.39^ only important for CCL21) (Figures 2, 3A). The tryptophan in top of TM2 (W114^2.60^), important for both ligands, is conserved in more than 80% of chemokines, while a non-polar aromatic residue is only present in less than 15% of non-chemokine class A receptors (*analyzed by GPCRdb.org*). The dependency of both CCL21 and CCL19 on Trp^2.60^ is interesting as it has been deemed important in both CCR5 and CXCR4 for initial chemokine-receptor interactions (23–25). The extracellular part of CCR7 was shown to be important for signaling of both chemokines with the N-terminal K50 and R54 residues being central for signaling (Figure 2B). In general, all mutations reached a plateau in their dose-response curves for CCL19 with efficacies similar to that on WT CCR7 (not shown), indicating that signaling changes could not be attributed to differential surface expression. Only R54A did not reach the efficacy of WT, yet this was due to severely impaired signaling as this mutant displayed surface expression levels similar to WT (Figure 3B). Looking at migration, a biologically more relevant readout, the effect the mutations had on G protein signaling (Figures 2A,B and Table 1) corresponded well with their effect on migration capacity of 300–19 pre-B cells stably expressing similar levels of either WT or mutant forms of CCR7 on the cell surface, shown by the three mutations L61A, W114A, and R54A (Figures 3B,C and Table 2). In a Transwell migration assay, three different concentrations of CCL19 and CCL21 were tested (1, 10, and 100 nM) where both ligands showed peak activity at 10 nM for WT CCR7. This peak was reduced significantly for the two mutations W114A^2.60^ and R54A^N−term^ both showing a major impairment of G protein signaling (Table 2). The L61A^1.35^ mutation reduced the peak at 10 nM slightly for CCL19, corresponding to the minor impact it had on G protein signaling for both ligands.

**Table 1:** Functional analysis of CCR7 mutations.

			**G protein**								**β-arrestin-2**			
**Residue**			**CCL21 efficacy**				**CCL19 potency**				**CCL19 efficacy**			
**Position**		**Number**	**Efficacy at 100 nM ± SEM**	**E_**mut**_**			**-logEC50 (M) ± SEM**	**F_**Mut**_**			**Efficacy at 100 nM ± SEM**	**E_**mut**_**		
N-term		K50A		**Dead**	*	*(3)*	8.5 ± 0.07	**13**	*	*(3)*	55.6 ± 2.6	**44**	*	*(5)*
		R54A		**Dead**	*	*(3)*	7.1 ± 0.07	**100**	*	*(3)*		**Dead**	*	*(5)*
		K57A	99 ± 6.4	**1**	*ns*	*(3)*	9.2 ± 0.12	**2**	*ns*	*(3)*	125.1 ± 13.6	**+25**	*ns*	*(3)*
TM1	1.35	L61A	44 ± 2.9	**56**	*	*(3)*	8.9 ± 0.13	**3**	*	*(3)*	36.2 ± 3.0	**64**	*	*(3)*
	1.39	Y65A	44 ± 1.5	**56**	*	*(4)*	9.0 ± 0.08	**2**	*ns*	*(3)*	74.8 ± 6.4	**25**	*ns*	*(5)*
TM2	2.53	F107A	76 ± 7.4	**24**	*ns*	*(3)*	8.9 ± 0.16	**2**	*ns*	*(3)*	149.1 ± 14.1	**+49**	*ns*	*(3)*
	2.60	W114A	32 ± 5.6	**68**	*	*(3)*	8.0 ± 0.09	**13**	*	*(3)*	41.7 ± 3.9	**58**	*	*(5)*
	2.63	S117A	68 ± 3.0	**32**	*ns*	*(3)*	9.5 ±+0.07	**1**	*ns*	*(3)*	139.4 ± 10.3	**+29**	*ns*	*(5)*
	2.64	A118S	67 ± 4.6	**33**	*ns*	*(3)*	9.7 ± 0.07	**2**	*ns*	*(3)*	133.5 ± 12.4	**+33**	*ns*	*(5)*
TM3	3.26	K130A	42 ± 3.5	**58**	*	*(3)*	9.0 ± 0.07	**7**	*	*(3)*	113.6 ± 7.8	**+14**	*ns*	*(3)*
	3.29	F133A	27 ± 4.5	**73**	*	*(4)*	8.7 ± 0.17	**1**	*ns*	*(3)*	73.6 ± 2.0	**26**	*	*(3)*
	3.33	K137A	43 ± 8.5	**57**	*	*(3)*	9.2 ± 0.29	**3**	*ns*	*(3)*	113.9 ± 4.4	**+14**	*ns*	*(3)*
TM4	4.60	E193A	45 ± 8.6	**55**	*	*(4)*	9.6 ± 0.17	**+3**	*ns*	*(3)*	119.7 ± 4.1	**+20**	*	*(4)*
ECL2		E205A	48 ± 5.3	**52**	*	*(4)*	9.2 ± 0.09	**3**	*	*(3)*	56.3 ± 4.7	**44**	*	*(3)*
		R209A	132 ± 8.8	**+32**	*	*(5)*	8.5 ± 0.14	**8**	*	*(5)*	54.3 ± 4.9	**46**	*	*(5)*
TM7	7.32	N305A	53 ± 3.6	**47**	*	*(3)*	8.6 ± 0.08	**4**	*	*(3)*	87.2 ± 2.7	**13**	*ns*	*(5)*
	7.36	Y308A	38 ± 3.4	**62**	*	*(3)*	9.1 ± 0.15	**2**	*ns*	*(3)*	98.9 ± 12.1	**1**	*ns*	*(3)*

**Figure 4:**
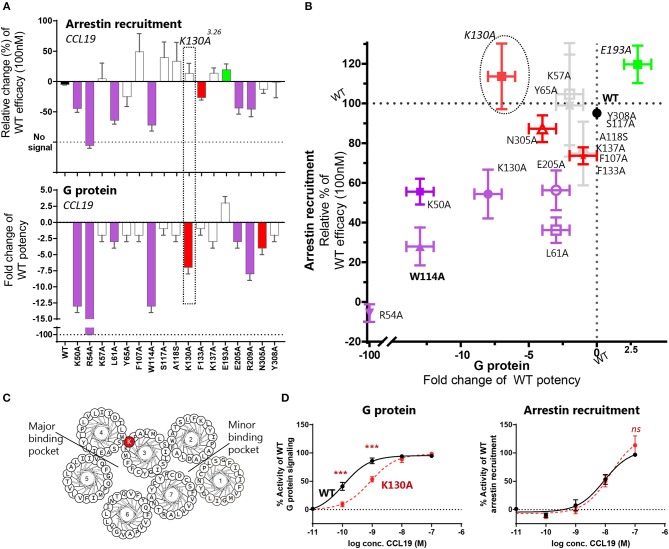
Mutagenesis study of CCR7 showing changes of CCR7 G protein or β-arrestin-2 recruitment upon CCL19-stimulation. **(A)** Barplot displaying change of β-arrestin-2 recruitment or G protein signaling by CCR7 mutations evaluated in the BRET based assays. Changes are displayed as relative change of efficacy at 100 nM CCL19, or fold change of CCL19 potency. Colors correspond to colors in **(B)**, where *purple* identifies mutations impairing both pathways, *green* refers to mutations improving signal, *red* refers to mutations only affecting one signaling pathway and *gray* identifies mutations with no impact. The mutation K130A^3.26^ selectively impairing G protein signaling is highlighted. **(B)** Scatterplot comparing the effect on signaling pathways. Mutations are plotted with their values from **(A)** and colored according to the description in **(A)**. **(C)** Helical wheel showing the location of K130^3.26^ in CCR7. **(D)** Dose-response curve of K130A^3.26^ stimulated with CCL19 in G protein signaling assay (left) and β-arrestin-2 recruitment assay (right). Statistical differences between K130A and WT curves are analyzed by ANOVA and significant differences identified by asterisks. Data are represented as mean value (±SEM) of at least three independent experiments performed in duplicates. To compensate for inter-assay variations data has been normalized to wildtype within each separate experiment before the collection of data. The *n* value of independent experiments can be found in Table 1.

Signaling values for CCR7 mutations in response to CCL21 or CCL19 analyzed in G protein signaling or β-arrestin-2 recruitment assays normalized to WT signaling. Data are presented in Figures 2, 4. Residue position refers to the location in CCR7 based on the nomenclature of Ballesteros-Weinstein (41). CCL21 G protein signaling and CCL19 β-arrestin-2 recruitment are compared to WT based on changes of efficacy at maximal agonist concentration used (100 nM), as saturation of signaling curves were not established. E_mut_ describes the percentage activity of WT, with WT activity reaching 100%. CCL19 G protein signaling is compared to WT based on fold change of potency, and F_mut_ describes the fold change of signaling potency compared to WT. “ns” refers to a non-significant change of signaling curve while “*” identifies significant changes between dose-response curves analyzed by ANOVA. Data are represented as mean values (±SEM) of several independent experiments performed in duplicates and always compared to the signaling values of CCR7 wild type run in parallel within the same experiments. To compensate for inter-assay variations data have been normalized to wild type within each separate experiment before the collection of data. The n number of independent experiments for each mutation is shown in brackets to the right of F_mut_ or E_mut_, (n). Bold values indicate main findings, italic values indicate statistical significance.

**Table 2:** Migratory capacity of CCR7 mutations.

	**CCL21 (10 nM)**		**CCL19 (10 nM)**	
**Mutation (position)**	**% Specific migration at 10 nM + SEM**		**% Specific migration at 10 nM + SEM**	
**WT**	5.1 ± 0.50		11.6 ± 1.58	
**R54A** *(N-term)*	0.3 ± 0.37	*	−0.1 ± 0.08	*
**L61A** *(1.35)*	4.0 ± 0.49	*	7.5 ±1.94	*ns*
**W114A** *(2.60)*	0.6 ± 0.45	*	1.0 ±0.66	*
**R209A** *(ECL2)*	10.6 ± 1.00	*	5.0 ± 0.55	*

*The specific migration of 300–19 pre-B-cells stably transfected with CCR7 wild type or mutant constructs in a Transwell migration assay is shown. Data are presented as the specific migration at 10 nM chemokine, at which concentration both CCL21 and CCL19 display peak activity, and values represent data presented in Figure 3. The specific migration is calculated as the percentage of specifically migrated cells relative to the input and shown as mean values (±SEM) from four independent experiments. Each mutant is compared to wildtype by ANOVA and asterisks identify significant differences, while “ns” refers to a non-significant change. Residue position refers to the location in CCR7 based on the nomenclature of Ballesteros-Weinstein, as shown in Table 1 (41). Bold values indicate main findings, italic values indicate statistical significance*.

### Mutations in ECL-2 Differentially Impair CCL21 and CCL19

With extracellular events at CCR7 seeming pivotal for differential chemokine interaction (as the core domain determined the difference between CCL21 and CCL19), it was interesting to identify that alanine substitution of R209 had the opposite effect on the two ligands (Figures 2B,D). The residue is located in the extracellular loop 2 (ECL-2), a receptor region of general importance for ligand recognition in GPCRs (42–44). In more details, R209 is located in ECL-2A, adjacent to the conserved cysteine (Cys-1) (Figure 3A). Upon the alanine substitution, an 8-fold decrease in the potency of CCL19 was observed (Figure 2D and Table 1). In contrast, signaling in response to CCL21 was improved with the signaling curve shifting significantly to the left (Figure 2D). In contrast to the positive R209 that appeared to be important for CCL19, the alanine substitution of the negatively charged E205^ECL−2^ seemed to have a more profound effect on CCL21 signaling than CCL19 (Figure 2), with a subtle 3-fold decrease of CCL19 potency, but a 50% decrease of CCL21 efficacy (Table 1). Together these studies suggest that CCL21 and CCL19 might interact differentially with the extracellular part of CCR7, especially ECL-2. The impact of the R209A mutation in CCR7 was also tested in the migration assay (Figure 3D and Table 2). Again, we observed that CCL19-mediated cell migration was selectively impaired by the R209A substitution, whereas the migration response to CCL21 was improved. Both ligands displayed peak activity at 10 nM, where the activity for CCL19 at this concentration was reduced to 42% (±20%) of WT while CCL21 was increased 2.5-fold compared to WT. This indicates that the differential ECL-2 interactions of the two chemokines are important for normal cell physiology.

### Top of TM3 Involved in Biased Signaling

**Figure 5:**
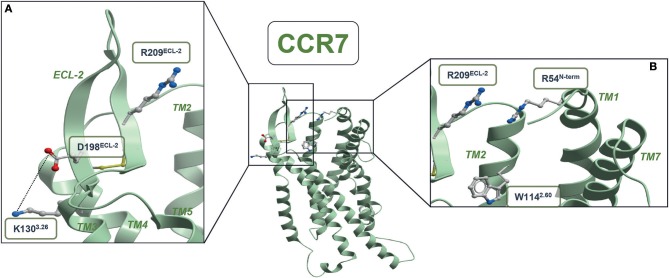
CCR7 homology model. **(A)** Structural link between ECL-2 and extracellular part of TM3 **(B)** entrance to the minor binding pocket. The CCR5-based homology model of CCR7 was built in the Molsoft ICM program (PDB ID 4MBS). R54^N−term^, W114^2.60^, K130^3.26^, C129^3.25^, D198^ECL−2^, R209^ECL2^, and C210^ECL−2^ are shown in stick representations. Dotted lines represent potential long-range charge-charge interaction between K130^3.26^ and D198^ECL−2^.

Through our mutagenesis study, we also wished to identify residues of selective importance for the two signaling pathways: G protein signaling and β-arrestin-2 recruitment. Since CCL21 hardly induces any β-arrestin-2 recruitment, we only screened the mutants for their impact on CCL19-stimulated β-arrestin-2 recruitment and compared this to their impact on CCL19-stimulated G protein signaling (Figures 4A,B and Table 1). The impact on β-arrestin-2 recruitment resembled the effect the mutations had on G protein signaling. One mutation K130A^3.26^, however, stands out. Alanine substitution of K130, located in the top of TM3 adjacent to the conserved cysteine (Cys+1) (Figure 4C), resulted in an impairment of G protein signaling without impairing β-arrestin-2 recruitment—if anything it increased β-arrestin-2 recruitment, although not significantly (Figure 4D). To understand the role of K130^3.26^ we constructed a homology model of CCR7 based on the X-ray structure of the human CCR5 chemokine receptor (40). From this model, we see that K130^3.26^ projects away from the binding pocket not likely to be involved in direct ligand-interactions (Figure 5). Interestingly, this residue is located in an area previously shown to be G protein-specific (20) or important for chemokine receptor signaling in general (42). Our model also allowed us to look into the positioning of W114 (the conserved Trp^2.60^), which projects from TM2 into the binding pocket. We found that R54 (highly important for both ligands and located at the TM1:N-terminal interface), projects across the binding pocket above W114, indicating that it could be involved in the process by which the ligands reach this residue. Our model further suggests that R54 might engage in unusual stacking with R209 in ECL-2, together forming a lid over the binding pocket (Figure 5B). Exploring our model further we found that K130^3.26^ is located near ECL-2, partly constrained by its proximity to the TM3-ECL-2 disulfide (C129^3.25^–C210^ECL−2^), and we propose that K130^3.26^ may make long-range charge-charge interactions with D198^ECL−2^ (Figure 5A). These suggestions are interesting taking our findings of R209A, also located in ECL-2, into account. K130^3.26^ and D198^ECL−2^ might function as a structural link between the R209^ECL−2^ situated on top of the binding pocket and the extracellular TM3-TM4 region shown to be important for signaling in other chemokine receptors (42). If true, it could indicate how the different ligand interactions with ECL-2 could be transmitted to different CCR7 signaling profiles of the two chemokines.

## Discussion

In the current study, we investigate the interactions displayed between the chemokine receptor CCR7 and its two ligands, CCL21 and CCL19, with the aim of understanding how they lead to differential signaling profiles of CCR7. Employing chimeric ligands, where the N-termini are swapped between CCL21 and CCL19, we identify the chemokine core domain to be the determining factor for their differential activation of CCR7, highlighting the importance of extracellular ligand-receptor events. Our studies also identify extracellular receptor interactions, in particular ECL-2, as important for differential ligand interactions.

### The Core Domains of CCL19 and CCL21 Determine Their Differential Signaling Profiles

For both chemokine chimeras employed in this study, we find that the core domain determines signaling outcome as the chimeras resembled the chemokine with which they share the core domain. The CCL19 chimera containing the N-terminus of CCL21, CCL19^CCL21N−term^, resembled CCL19 more than CCL21 in both G protein signaling and β-arrestin-2 recruitment assays, whereas the other chimera CCL21^CCL19N−term^ resembled CCL21. The slight decrease of potency when changing the N-terminus of CCL19 with that of CCL21 corresponds to a previous study describing that the only N-terminal residue within CCL19 crucial for its signaling at CCR7 is D7 which is also found within CCL21. Alanine substitution of the additional N-terminal residues only showed a minor impact on CCL19 signaling potency (45). This study indicated that D7 might be central for CCR7 activation in general, but that no N-terminal residue as such is involved in ligand specificity between CCL21 and CCL19, corresponding to the findings within our current study. These findings are also in accordance with another study showing that the viral US28 GPCR is quite independent of ligand N-terminal sequences for chemokine signaling but seems to simply rely on the N-terminus as a steric bulk in the orthosteric binding pocket (46).

Our current study does not disregard the importance of the N-terminus for signaling, we merely show that at CCR7 the specific N-terminal sequence of CCL21 and CCL19 does not dictate their biased signaling patterns. From the crystal structures of CXCR4:vMIP-II (28) and US28:CX3CL1 (27) it seems that different receptor-ligand structures display distinct orientations at the receptor-ligand interface with ligands positioned to interact with different receptor domains (32). Where one ligand can be positioned ideally to interact with ECL-2 though its 30s-loop (CX3CL1), corresponding to a CRS1 recognition, another ligand can be positioned to interact with ECL-2 through its N-terminus (vMIP-II), corresponding to a CRS2 recognition. A study of the core domain of CXCL8 showed that mutating an important sequence in the 30s-loop modulated the chemokine N-terminus and N-loop through intramolecular interactions which affected activation of its two receptors CXCR1 and CXCR2 differentially (47). Taken together these studies emphasize that the chemokine core can be important for directing the positioning of the chemokine N terminus and that the core domain may regulate the interactions displayed between chemokine and main receptor binding pocket. The structure of CCR5:CCL5 also shows that domains in the globular chemokine core dock deeper into the binding pocket than first assumed (26), showing that the core domain of CCL5 is important for more than a simple tethering function.

**Figure 6:**
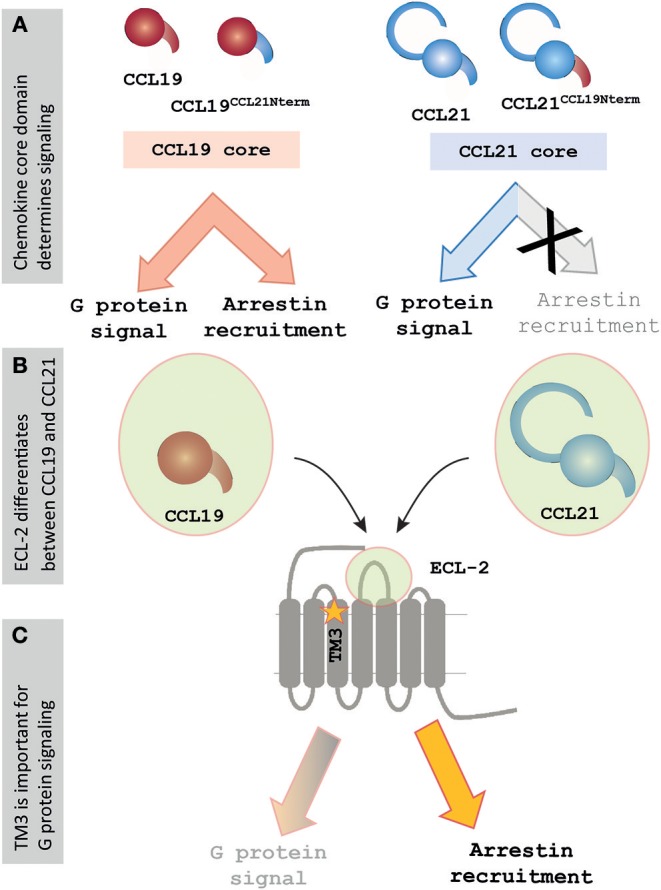
Signaling events at CCR7 rely on core domain interactions and ECL-2 of CCR7. **(A)** Chemokine cores determine differential signaling by CCL21 and CCL19. Chemokines containing the core of CCL19 display G protein signaling and β-arrestin-2 recruitment, whereas chemokines containing the core of CCL21 display G protein signaling but fail to recruit β-arrestin-2. **(B)** The current mutagenesis study of CCR7 shows that especially the extracellular events are important for chemokine signaling at CCR7, and the figure highlights ECL-2, which was found to play an important but differential role during interactions with the two chemokines. **(C)** The current study also found a lysine in TM3, K130^3.26^, to be important for regulating signaling, as the alanine substitution of this residue selectively impaired G protein signaling while not showing an effect on β-arrestin-2 recruitment upon CCR7 stimulation.

Differences of chemokine orientation resulting from interactions between the core domain and extracellular part of the receptor and the direct interactions displayed between the chemokine core domain and the receptor-binding pocket emphasize the importance of the core domains in chemokines. Together, this challenges the paradigm of chemokine signaling residing in the N-terminus. Docking of the N-terminus may be necessary for signaling initiation, but our studies of CCR7 indicate that it may be more in form of the presence of a steric bulb, as shown for US28 (46), and that the disparity in signaling patterns between CCL21 and CCL19 is instead attributed to differences in the chemokine core domains (Figure 6A).

### Identification of Differential Interaction Modes at CCR7

After finding the core domain to be pivotal for ligand differences it is interesting to identify a mutation in the extracellular part of CCR7 with differential impact on the two chemokines (Figure 6B). Thus, by mutating the centrally located charged R209 in ECL-2 to alanine an impairment of CCL19 signaling was observed concomitant with an improved signal of CCL21. The fact that chemokines acting at the same receptor may utilize the extracellular domains of the receptor differentially has also been described for CXCR2. Here the three ligands CXCL1, CXCL7, and CXCL8 showed differential interaction modes with the receptor's extracellular domains, with an example being that the receptor N-terminus was important for CXCL1 and CXCL8, but not CXCL7 (48). Studies with CCR5-CCR2 chimeric receptors also stress the importance of the extracellular domains for chemokine-mediated signaling, by showing that transferring all extracellular domains of CCR2 to CCR5 is both necessary and sufficient for ligand recognition and signaling initiation of CCR2-targeting chemokines (49).

Where CCR7 has previously been shown to display a CCL21-specific domain at the TM4-TM5 interface (7), no such CCL19-specific area has been identified. In our study, we only identified one residue R209^ECL−2^ [among 17 tested (Table 1)] to be selectively important for CCL19 signaling. In general, CCL19 signaling was predominately affected by mutations in the extracellular domains of CCR7 with alanine substitutions of K50^N−term^, R54 ^N−term^, and R209^ECL−2^ having a big impact on CCL19-signaling suggesting that important interactions between CCL19 and CCR7 are extracellular (Figure 6B). Following these lines it is interesting to note the potential stacking of R54^N−term^ and R209^ECL−2^ observed in our CCR7 homology model, forming a lid over the binding pocket. This lid might control the entrance of CCL21 and CCL19 into the binding pocket and engagement with the conserved Trp^2.60^, but with different contributions of these residues as both CCL21 and CCL19 are highly impaired by R54A, but CCL21 is not by R209A. The importance of ECL-2 is also seen within the group of lipid prostanoid GPCRs, such as the prostaglandin EP4 receptor (50), the prostanoid EP3 receptor (51) and the thromboxane A2 receptor (52), where the extracellular loop 2 is important for enclosing the ligand binding site. Similar to the current study, the ECL-2 of the prostanoid GPCRs was found to be central for ligand interactions as alanine substitution of central ECL-2 residues diminished ligand signaling in the EP4 and EP3 receptors (50, 51).

### Linking ECL-2 Interactions With CCR7 Signaling

A single residue located in TM3 was identified to be of potential importance for biased signaling at CCR7. Alanine substitution of this residue, K130^3.26^, impaired the G protein activation without affecting β-arrestin-2 recruitment (Figure 6C). This finding corresponds to biased areas identified in other class A GPCRs, such as GPR183, where TM3 was also identified as an important area for controlling G protein but not β-arrestin-2 signaling (20). It would be interesting if a distinct area of CCR7 could be identified as important for controlling β-arrestin-2 recruitment as seen with TM5 in CCR5 (19). Understanding the molecular mechanisms driving biased signaling is central for the development of more specific drugs targeting one among several signaling pathways to minimize the off-target effects when aiming at complex and promiscuous receptors such as in the chemokine system (4). The use of biased drugs is pursued not only within chemokine receptors but also in other non-chemokine GPCRs for better clinical outcomes (53, 54). Our homology model also suggested that K130^3.26^ may engage in long-range charge-charge interactions with D198^ECL−2^. On one hand, this is in line with observations from the chemokine receptor CCR8 where similar interactions between ECL-2 and an aromatic cluster in the TM4-TM5 region were identified to be important for receptor activity (42). On the other hand, it is possible to speculate whether the differential interaction of CCL21 and CCL19 with ECL-2 could be transmitted through D198^ECL−2^-K130^3.26^ resulting in a differential degree of receptor activation by the two ligands and, to some degree, explain the differential ligand signaling profiles.

## Conclusion

We identify the core domain of CCL21 and CCL19 to be a determining factor for their differential activation of CCR7, highlighting the importance of extracellular ligand-receptor events. The importance of extracellular receptor interactions are also underlined through our mutagenesis study, showing that especially ECL-2 of CCR7 seems important for differential ligand interactions. Furthermore, we propose a potential link between ECL-2 and the transmembrane domains, utilized differentially by the two chemokines. We do not disregard the importance of chemokine N-terminus docking into the receptor binding pocket, but our data suggest that the chemokine N-terminus in broad terms function as a steric bulb more than by specific residues being a determining factor of differential ligand signaling. Where small molecules are found to dock deep and be embedded in the receptor binding pocket, we suggest more focus should be given to extracellular receptor regions in order to understand the larger peptide chemokines, both in terms of ligand-receptor interactions, but also for future modulation of differential chemokine interactions and differential chemokine-mediated receptor signaling.

## Data Availability

The datasets generated for this study can be obtained from the authors upon request.

## Author Contributions

AJ and GH wrote the manuscript, designed and carried out experiments, analyzed and interpreted data. OL, ML, TF, EU, DL, and CV contributed to data acquisition and/or data interpretation. MR wrote the manuscript, designed experiments, analyzed, and interpreted data. All authors helped revise, and approve the manuscript, and agreed to be accountable for all aspects of the work.

### Conflict of Interest Statement

The authors declare that the research was conducted in the absence of any commercial or financial relationships that could be construed as a potential conflict of interest.
